# Cyclizine Toxicity and Misuse in an Adolescent With Eating Disorder and Depression: A Case Report

**DOI:** 10.7759/cureus.101873

**Published:** 2026-01-19

**Authors:** Ahmed Gamal Mohamed Abdelkhalk

**Affiliations:** 1 Paediatric Emergency Department, King's College Hospital, London, GBR

**Keywords:** adolescent mental health, antihistamine toxicity, cyclizine, drug misuse, intentional overdose, paediatric poisoning, pediatrics emergency

## Abstract

Cyclizine is a first-generation H1 antihistamine widely used for the treatment of nausea and motion sickness. In addition to its antiemetic properties, it has anticholinergic and central nervous system effects that may confer psychoactive potential. Although generally regarded as a low-risk medication, increasing evidence suggests a potential for misuse, particularly among adolescents and individuals with mental health disorders or substance-use vulnerabilities. Published data on cyclizine toxicity are limited, and paediatric reports of intentional overdose remain scarce. We report a case of intentional cyclizine overdose in a 14-year-old girl with underlying mental health disorders, highlighting the clinical presentation, management considerations, including serial electrocardiographic assessment, and safeguarding implications. This case underscores the need for increased clinician awareness of cyclizine misuse and its potential harms in the paediatric population.

## Introduction

Cyclizine is a first-generation H1 antihistamine with sedative and anticholinergic properties, commonly used in the management of nausea, vomiting, and dizziness associated with motion sickness [1,2]. The recommended oral dose for adults and adolescents is 50 mg taken approximately 30 minutes before travel, which may be repeated every four to six hours to a maximum of 200 mg in 24 hours [3]. Therapeutic plasma concentrations are reported to range between 0.1 and 0.25 mg/L [3,4].

Despite its widespread use and ready availability, often as an over-the-counter medication, cyclizine is generally perceived as a low-risk drug. Consequently, reports describing its toxic effects are limited, and fatalities attributed to cyclizine overdose are rare [5]. The existing literature consists largely of isolated adult case reports and small paediatric series, with very few contemporary reports [5,6]. Although antihistamines have been associated with cardiac conduction abnormalities in overdose, clinically significant cardiotoxicity attributable to cyclizine remains poorly characterised, particularly in paediatric patients. We present a case of intentional cyclizine overdose in an adolescent, highlighting the clinical features, management challenges, and associated mental health context [5,7].

## Case presentation

A 14-year-old girl presented to the paediatric emergency department approximately three hours after intentionally ingesting 33 tablets of cyclizine (50 mg each; total dose 1,650 mg). Her medical background was significant for major depressive disorder and a restrictive eating disorder with bulimic features, requiring long-term nasogastric tube feeding. She was under active follow-up by Child and Adolescent Mental Health Services (CAMHS).

Cyclizine had originally been prescribed for nausea related to enteral feeding intolerance; however, the patient had been self-administering the medication independently and without close supervision for a prolonged period. Following ingestion, she developed dizziness and marked agitation, associated with hypertension (148/98 mmHg), tachycardia (heart rate up to 166 beats per minute), tachypnoea (36 breaths per minute), and a subjective sensation of rapid, uncontrolled breathing. She subsequently disclosed the ingestion to her parents, who brought her immediately to the emergency department.

In the week preceding presentation, the patient experienced a deterioration in her mental health, characterised by increasing emotional distress and behavioural dysregulation. Inpatient psychiatric admission had been recommended by her treating psychiatrist, but was declined by the patient. On the day of presentation, she deliberately ingested cyclizine tablets from her own supply with suicidal intent.

On arrival, she was alert but visibly agitated. Initial management consisted of supportive care with continuous cardiac monitoring, pulse oximetry, and supplemental oxygen. Intravenous access was secured, and baseline investigations were obtained, including full blood count, renal profile, liver function tests, blood glucose, creatine kinase, and venous blood gas analysis (Tables 1, 2).

**Table 1:** Laboratory investigations on admission. All laboratory values were obtained on admission. Results are presented with corresponding reference ranges.

Parameter	Result	Reference range
Haemoglobin	127 g/L	120–150
White cell count	9.9 ×10⁹/L	4.0–10.0
Platelet count	306 ×10⁹/L	150–400
Sodium	144 mmol/L	133–146
Potassium	3.5 mmol/L	3.5–5.0
Urea	4.9 mmol/L	2.5–6.5
Creatinine	62 µmol/L	40–80
Alanine transaminase (ALT)	24 U/L	10–35
Alkaline phosphatase	116 U/L	57–254
Creatine kinase (CK)	77 IU/L	25–200
Glucose	4.9 mmol/L	3.5–7.8

**Table 2:** Comparison of initial and repeat venous blood gas results. Venous blood gas (VBG) analysis demonstrating an initial respiratory alkalosis with subsequent improvement on repeat testing following supportive management. pCO_2_: partial pressure of carbon dioxide; HCO_3_: bicarbonate.

Parameter	Initial VBG	Repeat VBG	Reference range
pH	7.51	7.45	7.35–7.45
pCO₂ (kPa)	3.5	4.0	4.7–6.0
HCO₃⁻ (mmol/L)	23.7	22.8	22–26
Base excess	−2.0	−2.8	−2 to +2
Lactate (mmol/L)	2.2	2.0	<2.5
Oxygen saturation (%)	87.8	95.2	>94

Initial results demonstrated respiratory alkalosis consistent with hyperventilation, which improved on repeat testing following supportive management.

Electrocardiographic findings

**Figure 1:**
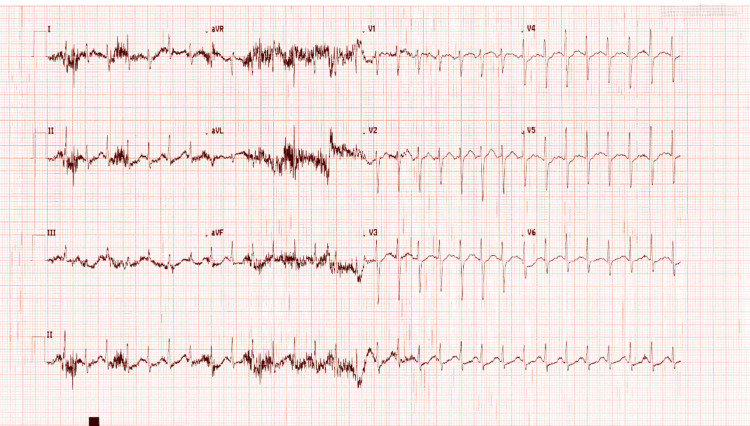
Initial 12-lead electrocardiogram demonstrating sinus tachycardia following cyclizine overdose. An initial 12-lead electrocardiogram obtained on presentation showed sinus tachycardia with a ventricular rate exceeding 170 beats per minute. QRS complexes were narrow, and the corrected QT (QTc) interval was within normal limits. Non-specific ST–T wave changes and prominent motion artefact were present, consistent with agitation and anxiety. No evidence of QRS widening, QT prolongation, or malignant arrhythmia was observed.

Serial electrocardiographic assessment was performed in view of the significant tachycardia following cyclizine overdose. The initial 12-lead ECG obtained on arrival demonstrated sinus tachycardia with a ventricular rate exceeding 170 beats per minute, narrow QRS complexes, and a normal corrected QT (QTc) interval. Non-specific ST-T wave changes and prominent motion artefact were present, consistent with agitation and anxiety. There was no evidence of QRS widening, QT prolongation, or malignant arrhythmia, reducing concern for acute cardiotoxicity (Figure 1).

**Figure 2:**
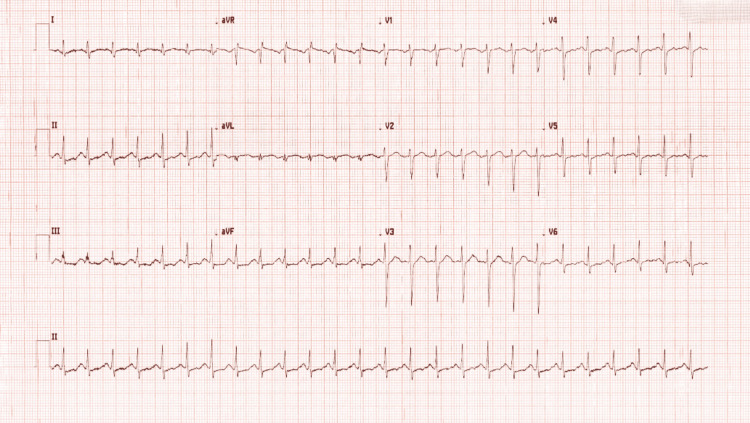
Repeat 12-lead electrocardiogram demonstrating improvement in sinus tachycardia following supportive management. Repeat 12-lead electrocardiogram obtained after supportive management, including intravenous fluids and benzodiazepine administration, showed persistent but improved sinus tachycardia with clearer P-wave morphology preceding each QRS complex. QRS duration and corrected QT (QTc) interval remained within normal limits. No evidence of supraventricular arrhythmia, QRS widening, or QT prolongation was observed. Reduced motion artefact correlated with clinical stabilisation and improvement in agitation.

A repeat ECG performed approximately one hour later, following supportive management, including intravenous fluids and intravenous lorazepam for agitation, demonstrated persistent but improved sinus tachycardia with clearer P-wave morphology preceding each QRS complex (Figure 2). PR, QRS, and QTc intervals remained within normal limits. Although automated ECG interpretation suggested a possible supraventricular tachycardia, manual review confirmed sinus tachycardia without atrial flutter or other supraventricular arrhythmia. The improvement in heart rate and reduction in artefact correlated with clinical stabilisation and resolution of agitation. Overall, the ECG findings were consistent with a physiological response to anxiety and hyperventilation rather than primary cyclizine-induced cardiotoxicity.

Following benzodiazepine administration, the patient’s blood pressure stabilised, and her tachypnoea gradually improved. She was admitted to the high-dependency unit for continued observation and supportive care. No further complications were observed, and serial monitoring remained reassuring. After approximately 12 hours of observation, she was deemed medically fit for discharge and was discharged home with comprehensive safeguarding measures and planned follow-up with CAMHS, her general practitioner, and her treating psychiatrist.

## Discussion

Cyclizine addiction and potential for misuse

Cyclizine is increasingly recognised to have misuse and addiction potential, despite being widely perceived as a low-risk antihistamine. A Coroner’s Prevention of Future Deaths report highlighted a lack of awareness among healthcare professionals regarding cyclizine abuse, noting that it is not routinely included in standard toxicology screening panels. This limited recognition may contribute to the underestimation of risk at the point of prescribing or supply [7-9].

Misuse of cyclizine has been reported for its sedative, euphoric, and hallucinogenic effects. It is most commonly taken orally, but in severe cases, it may be administered intravenously following crushing of tablets. Cyclizine acts as a central nervous system depressant, and its effects are potentiated when combined with other CNS depressants, including alcohol, benzodiazepines, and opioids [10-12].

Concomitant use with opioids, particularly methadone, has been well described, with users reporting enhanced psychoactive effects. At higher doses, cyclizine’s anticholinergic properties may induce hallucinations. Co-ingestion with alcohol is particularly hazardous, as cyclizine’s antiemetic effects may suppress vomiting and increase the risk of severe alcohol toxicity [12,13].

The World Health Organization defines psychoactive substances as agents that affect mental processes such as perception, consciousness, cognition, mood, or emotions. Within this framework, cyclizine meets the criteria for a psychoactive drug. Epidemiological data further support concern, with an increasing trend in fatalities involving sedating antihistamines, including cyclizine, reported in England over recent decades [11,14,15].

Discussion

Antihistamines are among the most commonly misused over-the-counter medications in the United Kingdom. Cyclizine misuse has been described across a range of populations, including adolescents and individuals with substance-use disorders, via both oral and intravenous routes. Intravenous misuse typically involves crushed tablets and is frequently associated with concurrent opioid use [6,7].

The principal driver for cyclizine misuse appears to be its euphoric or stimulant effect, often described as a transient “high” lasting 30-120 minutes and followed by a pronounced “low”. Some habitual users report suicidal ideation following resolution of these psychoactive effects. Cyclizine may also potentiate opioid-related psychoactive effects, which may explain its frequent co-administration in substance-using populations [6,7,10].

Chronic misuse has been associated with craving, tolerance, and a non-specific withdrawal syndrome, predominantly psychological but occasionally physical. Reports of aggressive behaviour, seizures, and violent acts have been described in the context of cyclizine abuse effects that are not observed with therapeutic use [5,10].

Although antihistamines in overdose have been associated with cardiac conduction abnormalities, this case demonstrated no evidence of clinically significant cardiotoxicity, with serial electrocardiograms showing sinus tachycardia without QT prolongation or malignant arrhythmia. The observed tachycardia and respiratory alkalosis were consistent with anxiety and agitation rather than direct cardiac or metabolic toxicity. This highlights the importance of careful clinical assessment and monitoring, as the absence of severe cardiotoxic features does not preclude the need for observation and safeguarding in intentional overdose [11,15].

This case illustrates the heightened vulnerability of adolescents with underlying mental health disorders to medication misuse, particularly when medications are readily accessible and perceived as benign. It reinforces the need for careful prescribing, supervision, and regular medication review in young people with complex psychological and medical needs [6-8].

## Conclusions

Cyclizine is an effective and generally well-tolerated antiemetic when used appropriately; however, its psychoactive properties confer a real potential for misuse, dependence, and toxicity. This case demonstrates that intentional overdose in adolescents may present with significant agitation and physiological disturbance without overt cardiotoxicity, while still requiring careful monitoring and multidisciplinary management. Healthcare professionals should exercise vigilance when prescribing cyclizine, ensuring adherence to licensed indications and dosing, alongside patient and family education. Robust safeguarding measures and regular medication review are essential in young people with complex mental health needs. Increased clinician awareness may help reduce preventable harm and associated morbidity.
